# Does promotion foster career sustainability? A comparative three-wave study on the role of promotion in work stress, job satisfaction, and career-related performance

**DOI:** 10.1007/s10775-024-09694-3

**Published:** 2024-08-12

**Authors:** Shagini Udayar, Ieva Urbanaviciute, Christian Maggiori, Jérôme Rossier

**Affiliations:** 1 Swiss National Centre of Competence in Research LIVES, University of Lausanne, Lausanne, Switzerland; 2 Institute of Psychology, University of Lausanne, Lausanne, Switzerland; 3 Institute of Psychology, Vilnius University, Vilnius, Lithuania; 4 School of Social Work Fribourg – HES-SO, Fribourg, Switzerland

**Keywords:** Promotion, Career sustainability, Well-being

## Abstract

The present study investigates the role of promotion in employees’ happiness (job satisfaction), health (work stress), and career-related performance (perceived employability and career prospects). Positive and negative changes in the above-mentioned career sustainability indicators were investigated over a 2-year period. The promotion subsample (*n* = 128) was compared with a matched sample of non-promoted employees (*n* = 150). We also tested the role of gender in responding to a promotion. The findings suggest that the promotion may have equivocal effects on employees’ happiness, health, and career-related performance over time, and therefore does not foster their career sustainability.

## Résumé

La présente étude examine le rôle de la promotion dans le bonheur des employés (satisfaction professionnelle), la santé (stress au travail) et la performance liée à la carrière (employabilité perçue et perspectives de carrière). Les changements positifs et négatifs dans les indicateurs de durabilité de carrière mentionnés ci-dessus ont été étudiés sur une période de deux ans. L'échantillon de promotion ( *n* = 128) a été comparé à un échantillon apparié d'employés non promus ( *n* = 150). Nous avons également testé le rôle du genre dans la réponse à une promotion. Les résultats suggèrent que la promotion peut avoir des effets équivoques sur le bonheur, la santé et la performance liée à la carrière des employés au fil du temps, et ne favorise donc pas leur durabilité de carrière.

## Abstract (de)

Die vorliegende Studie untersucht die Rolle der Beförderung für das Glück der Mitarbeiter (Arbeitszufriedenheit), die Gesundheit (Arbeitsstress) und die karrierebezogene Leistung (wahrgenommene Beschäftigungsfähigkeit und Karriereaussichten). Positive und negative Veränderungen in den oben genannten Indikatoren für die Karrierenachhaltigkeit wurden über einen Zeitraum von zwei Jahren untersucht. Die Beförderungs-Teilstichprobe ( *n* = 128) wurde mit einer passenden Stichprobe von nicht beförderten Mitarbeitern ( *n* = 150) verglichen. Wir haben auch die Rolle des Geschlechts bei der Reaktion auf eine Beförderung getestet. Die Ergebnisse deuten darauf hin, dass die Beförderung möglicherweise zweideutige Auswirkungen auf das Glück, die Gesundheit und die karrierebezogene Leistung der Mitarbeiter im Laufe der Zeit haben kann und daher ihre Karrierenachhaltigkeit nicht fördert.

## Resumen

El presente estudio investiga el papel de la promoción en la felicidad de los empleados (satisfacción laboral), salud (estrés laboral), y rendimiento relacionado con la carrera (empleabilidad percibida y perspectivas de carrera). Se investigaron los cambios positivos y negativos en los indicadores de sostenibilidad de carrera mencionados anteriormente durante un período de dos años. La submuestra de promoción ( *n* = 128) se comparó con una muestra emparejada de empleados no promovidos ( *n* = 150). También probamos el papel del género en la respuesta a una promoción. Los hallazgos sugieren que la promoción puede tener efectos equívocos en la felicidad, la salud, y el rendimiento relacionado con la carrera de los empleados a lo largo del tiempo, y por lo tanto no fomenta su sostenibilidad de carrera.

## Introduction

The current literature in work and career psychology frequently focuses on the impact of unequivocally negative professional events when investigating factors that may hinder employee well-being, career development, and thus career sustainability (Gerhardt et al., 2021; Lee et al., 2018). Major effort has been put into understanding the detrimental consequences of various stressors and career shocks related to organizational change, job insecurity, or job loss (e.g., Griep et al., 2016; Klehe et al., 2012; Lee et al., 2018), whereas relatively little attention has been paid to the seemingly beneficial events, such as promotion, on employee functioning from a career perspective. Nevertheless, recent studies have shown that positive events, and especially positive work events, are important to consider when studying career sustainability (e.g., Udayar et al., 2021).

A promotion is usually seen as a positive professional event that significantly alters one’s career path (Akkermans et al., 2018). Getting promoted is considered beneficial to the employee’s well-being because it is often accompanied by feelings of satisfaction and happiness (Pergamit & Veum, 1999). However, despite these positive effects, a promotion may also have some unexpectedly negative consequences (Johnston & Lee, 2013). After being promoted, the individual may encounter, for instance, new explicit or implicit demands in terms of productivity, supervisory responsibility, and working time. As a result, employees have to cope with new challenges and tasks for which they were not necessarily prepared and trained, which may affect job satisfaction and other work outcomes. An increase in seniority can also have a negative spillover effect, for example, implying worsened health (Boyce & Oswald, 2012). Hence, while considered a normatively positive career event, a promotion may turn out to have some undesirable consequences.

To date, most of the research effort has been dedicated to identifying the predictors of getting a promotion or achieving a career success in general rather than investigating its psychological after-effects. The outcomes of a promotion remain incompletely understood, especially from a psychological and career development perspective. The present study thus contributes to existing literature in the filed by attempting to address this gap. Drawing on the career sustainability framework (De Vos et al., 2020) and employing a comparative approach (i.e., conducting comparisons by promotion status, and additionally, by gender), we sought to inspect potential positive and negative changes in the key aspects of employees’ sustainable career development as a response to being promoted at work. Specifically, our study focused on the indicators of work-related health (i.e., perceived work stress), happiness (i.e., job satisfaction), and career-related performance (i.e., perceived employability and career prospects) over 2 years spanning from the pre-promotion to the post-promotion period. As a result, our findings offer new insights into the dynamics of employees’ promotion experiences that are relevant both in research and practical areas.

### The career sustainability theoretical framework: the role of events and time

According to the career sustainability framework (De Vos et al., 2020), a sustainable career involves a dynamic interrelation between the person, context, and time, with the level of career sustainability being measured by the individual’s health, happiness, and performance. It means that career sustainability should be understood both from a systemic perspective (the influence of multiple contexts and events) and a dynamic perspective (the influence of changes over time).

Research on career sustainability has gained increasing importance in the recent years (Van der Heijden et al., 2020). However, the role of life events, and especially work-related events, have been only scarcely studied in relation to career sustainability (e.g., Udayar et al., 2021, 2024), although some studies have examined the relation between career chance events and career development (see Kim, 2022 for a literature review) or career shocks and career sustainability (e.g., Hakanen et al., 2021; Sulbout & Pichault, 2022; Visentini et al., 2023). A recent study focused on the role of a broad spectrum of life events on all three indicators of career sustainability (Udayar et al., 2021), highlighting that work events are important to consider when studying career sustainability as there is evidence for spillover effects from work to life. Interestingly, experiencing positive work events seems to foster career sustainability, and these effects seem to be stronger than the negative effect of negative work or nonwork life events on health, happiness, and productivity. Such findings hint at the importance of positive (versus negative) work events in understanding career sustainability, and thus call for more research in this direction.

Because individuals are facing more frequent and varied transitions, we need to better understand how changes triggered by life or work events can impact one’s level of health, happiness, and career-related performance over the life course. Having a clearer picture of those relations may undoubtedly help career counselors as well as human resource management professionals to better guide people whose career sustainability is threatened. A career could be considered sustainable when, despite facing negative or positive events and changes, people experience continuity and meaning in their career paths, and thus remain healthy, happy, and productive throughout the life course. An event could happen in different contexts (work, leisure, family, etc.) and may interact with the key background characteristics of the person (such as gender) to significantly impact the sustainability of their career. The time dimension also plays a crucial role here by unraveling the dynamics of the relation between an event and the three indicators of career sustainability. Indeed, some events might have immediate consequences on health, happiness, and productivity while other consequences might appear after a longer period (De Vos et al., 2020). Therefore, adopting a longitudinal perspective is crucial for determining whether an event fosters or hinders career sustainability. In short, integrating the events and time dimensions while considering some characteristics of the person can inform about how (un)sustainable career development unfolds over the life course.

### Promotion, a positive work event or not so much?

Promotion is a focal event in one’s career trajectory that implies a significant upward shifting in the organizational hierarchy bringing greater responsibilities, power, and privileges (Pergamit & Veum, 1999). Because getting a promotion is a way to advance one’s career by achieving higher status, the literature often describes it as an indicator of career success (Ng et al., 2005; Spurk et al., 2018) and as a favorable career event (Akkermans et al., 2018; Seibert et al., 2013). Drawing on this positive conceptualization, many studies have shown interest in how to promote one’s career success by identifying the predictors of a promotion (e.g., Judge et al., 1995; Ng et al., 2005). For instance, a meta-analysis of Ng et al. (2005) has revealed that factors such as human and social capital, training and skill development opportunities, sociodemographic variables, and individual differences predict the chances of getting a promotion.

Although promotion is considered as a desired work outcome, it inevitably brings changes to employees’ working routine, re-shaping their jobs, and leading them to mobilize new resources and skills to face new and more frequent demands (e.g., Asselmann & Specht, 2023). Despite the desire to obtain a promotion, workers are not necessarily aware about or prepared to manage these new demands. Indeed, insecurity and turbulence in the labor market have reduced career advancement opportunities. Given temporary, short-term contracts and an increased number of inter-organizational job transitions accompanied by fewer intra-organizational job transitions (e.g., Kattenbach et al., 2014), it may be more difficult to predict when and under which conditions one can get a promotion. Moreover, in some organizations, opportunities for internal advancement are curtailed by downsizing (Lyons et al., 2015). In addition, due to the pressure by organizations, some workers have to accept a promotion that they may not particularly want (Kim, 2019). All these aspects suggest that a promotion may bring both positive and negative experiences that need to be better understood.

### The consequences of a promotion

While the predictors of a promotion have been well explored and identified, its consequences have received somewhat less attention. Notably, the existing evidence on the after-effects of being promoted is rather mixed. On the one hand—and rather expectedly—a few positive consequences have been identified. For example, a promotion has been associated with wage increase, higher status in the company, and new opportunities for development (e.g., Gibbons & Waldman, 2006; Pergamit & Veum, 1999), as well as with lower probability of withdrawal and turnover (e.g., Stumpf, 2014). Other positive effects also comprise career success expectancy, higher job security, perceived procedural justice, better human capital assessments by the supervisor, and higher core self-evaluations (Armstrong-Stassen, 2003; Stumpf & Tymon, 2012). On the other hand, some negative consequences have been reported as well. For instance, promotion has been found to negatively affect employee mental health in terms of stress, anxiety and depression, and self-rated health (Boyce & Oswald, 2012; Johnston & Lee, 2013; Nyberg et al., 2017). There is also some recent evidence that starting a leadership position may compromise specific facets of affective subjective well-being (see Asselmann & Specht, 2023). It is also notable that the outcomes of the promotion may be time varying. Johnston and Lee (2013) found that getting a promotion was positively related to a host of positive outcomes, such as job security, pay perceptions, and job satisfaction, but in the short term only. In contrast, negative aspects such as work stress remained high even 2 years after getting promoted. Moreover, they found a stronger effect of promotion on job attributes, health, and well-being after 6–12 months, which points out the importance of studying the outcomes of a promotion for at least 1 year.

Given that findings regarding long-term consequences of a promotion are scarce, there is a need for further research investigating how a promotion may affect not only employee work-related health and happiness but also career-related performance such as perceived employability and career prospects, which has been to our knowledge not studied using a longitudinal perspective to date. Such research may ultimately inform us on the role of promotion for a sustainable career development.

### The role of promotion in work stress and job satisfaction

Subjective well-being at work refers to employees’ evaluation of their workplace experiences in both cognitive and affective terms (Warr, 2013). Work stress and job satisfaction, respectively, are often used as the negative and the positive indicators of subjective well-being at work (Warr, 2013). Work stress results from the job demands that are appraised as taxing or exceeding resources and thus endangering employee well-being (Lazarus & Folkman, 1984). Job satisfaction, on the contrary, refers to a positive emotional state resulting from the evaluation of one’s job experience (Locke, 1969) and is also considered an indicator of subjective career success (Nicholson & De Waal-Andrews, 2005). Hence, it is not surprising that previous promotion studies have mainly considered these indicators. Where job satisfaction is concerned, most of these studies have found a positive effect of promotion (e.g., Francesconi, 2001; Pergamit & Veum, 1999), although this positive effect may be temporary. Indeed, Johnston and Lee (2013) have provided first evidence that promotion increases job satisfaction on a temporary basis only, whereas in the long run, the levels of job satisfaction turn back to the pre-promotion level. In addition to this, they have found that work stress seems to increase and remains at the increased level after the promotion. Furthermore, Asselmann and Specht’s (2023) study suggests that promotion to a leadership position may be related to increased levels of anger. These findings are particularly important as they imply that a promotion may have some short- and long-term negative effects that contradict the normatively positive expectations related to it. Hence, we can imply that promotion is a significant positive event that potentially triggers a momentary (i.e., not persistent) increase in the baseline level of job satisfaction but may also simultaneously lead to a long-term increase in the baseline level of work stress. Consequently, promotion will not necessarily foster a long-term career sustainability in terms of health and happiness indicators. On the basis of the above, we formulate the following hypothesis: Promotion has equivocal consequences on job satisfaction and work stress as indicators of sustainable career development (*H1*). On the one hand, promoted employees will report a higher level of job satisfaction without a persistent linear increase in it from T0 (1 year before the promotion year) to T2 (1 year after the promotion year), while job satisfaction will remain stable among the non-promoted employees over time (*H1a*). On the other hand, the promotion sample will report a higher and more persistent linear increase in work stress from T0 to T2 as compared with the non-promotion sample (*H1b*).

### Promotion and career-related performance

Perceived employability refers to individuals’ perceptions of their potential in the labor market (van Harten et al., 2022). According to the career sustainability framework, high employability could be used as an indicator of productivity/performance; hence, together with health and happiness indicators, it defines the sustainability of contemporary careers (Baumer de Azevedo et al., 2022; De Vos et al., 2020).

While employability has caught substantial attention in the work and career psychology literature (see Fugate et al., 2021 for a review), empirical research on the potential interrelation between promotion and employability is quite limited. Van der Heijden et al. (2009) found a positive relation between promotions and perceived employability. A more recent study pointed out the mediating role of perceived external employability in the relation between international work experience and promotion (Andresen et al., 2022). In this study, perceived external employability, defined as one’s perceived opportunities to get future job with another employer (Vanhercke et al., 2014), was considered as a personal resource that helped to attain career success. Thus, international work experience increased perceived external employability, which in turn increased the number of promotions the person was awarded. However, no causality effects were tested in those studies; therefore, there is no evidence that perceived employability is necessarily an antecedent of promotion history. In fact, one could argue that the reverse may equally be true. Getting a promotion is likely to increase perceived opportunities in the labor market. Therefore, a promotion may enhance one’s capabilities, especially transferable skills, increase one’s positive self-evaluations, and give confidence toward employability. Nelissen et al. (2017) found a positive impact of promotions on perceived external employability, which in turn had a positive impact on turnover intention. Recently, Ali and Mehreen (2022), found that positive career shocks have an impact on proactive career behaviors through perceived employability, which shows that an unexpected positive work event such as promotion could increase the level of one’s perceived employability. Moreover, Blokker et al. (2019) showed that positive career shocks strengthen the positive link between career competencies and perceived external employability. Although the impact of work events on perceived employability has been established in all three studies, they did not use a longitudinal perspective to analyze this relation. Recently, a study called for more research investigating the relation between major life events including work events and employability (De Lange et al., 2021).

According to the conceptualization of resources as defined by the conservation of resources theory (COR; Hobfoll, 1989), a promotion can be considered as a key resource leading to positive outcomes, both in the short and long run, by facilitating the accumulation of further resources. Indeed, a promotion implies higher employment status, which is both a resource in itself and a means for generating more resources and eventually gaining a better position in the labor market. Therefore, we expect an increase in perceived employability from T0 to T2 among the promoted employees, while perceived employability will remain stable among the non-promoted ones (*H2*).

Concerning career prospects, which refer to individuals’ perception of their career progression possibilities in the future (Urbanaviciute et al., 2019), one study showed that positive work events had a positive effect on perceived career prospects in both the short and long run (Udayar et al., 2021). However, it has never been studied in relation to promotion history to our knowledge. Nevertheless, similarly to perceived employability, we could expect that getting a promotion will increase perceived possibilities of career progression in the future by giving confidence in one’s competencies. In fact, by getting a promotion, the person gains a better position in the labor market (objectively or subjectively), further boosting their future perceived career prospects. Conversely, not getting any promotion should decrease the level of perceived career prospects, which may act as a barrier and hinder career development and sustainability. Again, on the basis of the COR theory, we expect an increase in perceived career prospects from T0 to T2 among the promoted employees, while perceived employability will remain stable among the non-promoted ones (*H3*).

### Does gender play a role?

Arguably, while a life or career event may strongly affect some people, their effect on others may be only negligible. This raises the question of factors (i.e., boundary conditions) that determine the strength and valence of the outcomes of a given event. Career sustainability framework highlights the need of considering sociodemographic characteristics to understand the dynamic relation between the person and context over time, and some recent studies showed the importance of gender differences in examining sustainable career paths (e.g., Dlouhy & Froidevaux, 2022; Udayar et al., 2024). Therefore, in this study, as a subsidiary aim, we propose to investigate how gender might determine the effects of promotion on employee’s work-related well-being and career-related performance, and thus on their career sustainability.

Previous findings on gender differences have revealed that women possibly experience more negative consequences after the promotion than men (Francesconi, 2001; Nyberg et al., 2017). In the study by Nyberg et al. (2017), women but not men reported a decrease in self-rated health after a promotion. Francesconi (2001) also demonstrated that the positive effect of a promotion on job satisfaction was greater for men than for women, whereas Lup (2018) found a decline in job satisfaction among women promoted to higher-level management positions. This is in line with the ideas developed in the gender literature that women in higher positions are more likely to have negative experiences because of negative stereotypes about women in managerial roles, exclusion from the high-status network, and/or due to higher work-life conflict threat (e.g., Heilman et al., 1995; Lup, 2018; McGuire, 2002). However, findings on gender differences in reacting to a promotion are not completely consistent. For instance, Johnston and Lee (2013) showed that in some cases, compared with women, men were more likely to experience negative consequences of a promotion, which asks for a further investigation of gender aspects. Our last research question is the following: Does gender affect employees’ level of health, happiness, and career-related performance after a promotion, and in which ways? (*RQ1*)

## Method

### Procedure and participants

The data for the current study were drawn from a longitudinal Professional Paths survey conducted annually from 2012 to 2018 within the Swiss National Center of Competence in Research LIVES (NCCR LIVES). For this survey, a representative sample of adult individuals (aged 25 to 55 years) living in Switzerland was drawn on the basis of a random sample from the Swiss Federal Statistics Office and the State Secretariat for Economic Affairs. The original dataset included both employed and unemployed participants from the German- and French-speaking areas of Switzerland representing a variety of occupational sectors. At different data collection points, the sample size ranged from 2469 to 1075 participants. More details on the methodology of this survey can be found in Maggiori et al. (2016). The survey materials data are archived in the SWISSUbase data repository and are available upon request (https://www.swissubase.ch/en/catalogue/studies/12734/15746/overview).

For the purposes of the present study, we only used data from employed individuals. First, we identified individuals who had been promoted at least once during the survey period (promotion was measured annually as a self-reported variable). On the basis of this information, we subsequently extracted sets of three consecutive waves of data for each promoted individual. These datasets could be from any period of the study provided that they satisfied the criteria for drawing a substantial sample of participants who had experienced a promotion at the middle timepoint. As a result, the promotion subsample (*n* = 128, mean age 41.02, SD 8.70) included respondents that experienced a promotion at the second of the three measurement points (i.e., no promotion at T0, promotion experience at T1, no promotion at T2). If two such patterns occurred for the same participant within the study period, only the first one was considered. On the basis of the same dataset, a second, non-promotion subsample (*n* = 150, mean age 41.76, SD 7.88) was randomly drawn from a pool of participants with no promotion experience at any given point of the study (*N* = 990). To make a comparison between the two subsamples possible, the non-promotion subsample was matched with the promotion subsample in terms of age, gender, having minor children, and the waves of the study from which the three measurements were taken.

In sum, both samples were composed as follows: 50.8% of the promoted individuals and 50.7% of the non-promoted individuals were women, 43.9% of the promoted ones and 42% of the non-promoted ones were married during the study period (from 1 year before to 1 year after the promotion), and one-third of individuals in both samples had children under 18 years old throughout the study period. Regarding their professional situation, some were employed for less than 1 year in the organization at T0, while others were employed for more than 10 years in the same organization. In the promotion sample, 68% were employed full time at T0 (63.2% of them were men), while 24.3% had a substantial part-time job (90.3% of them were women), and 2.3% of women had a marginal part-time job and 5.5% did not indicate their work rate. In the non-promotion sample, 61.3% were employed full time at T0 (66.3% of them were men), while 33.3% had a substantial part-time job (86% of them were women), and 1.3% of women had a marginal part-time job and 4% did not indicate their work rate.

### Measures

The variables of interest in the current study were measured as part of a large longitudinal survey. For this reason, most of the outcome indicators that denote specific, and thus quite narrow, constructs (except those assessing work stress) were based on single-item measures, as detailed below. Recent research, such as a study by Matthews et al. (2022), showed that narrow concepts can be measured reliably with single-item measures.

*Work stress (GWS)*. Work stress was assessed with the General Work Stress Scale developed by De Bruin and Taylor (2005). It is a nine-item scale that provides a measure of the level of stress caused by work. Responses were indicated on a five-point scale (1, never to 5, always). A sample question was: “Does work make you so stressed that you wish you had a different job?” Cronbach’s alphas were: 0.86 at T0, 0.87 at T1, and 0.89 at T2.

*Job satisfaction (JS)*. To evaluate job satisfaction, a one-item measure was developed for the aims of the Professional Paths survey. It asked participants to evaluate the overall satisfaction with their current job (“How satisfied are you with your current job?”), using a four-point response scale (1, not satisfied at all to 4, very satisfied).

*Perceived employability (PE)*. It was measured with a single item that corresponds to other single-item measures applied in previous studies (e.g., De Cuyper et al., 2010). The participants were asked to rate their perceived difficulty of finding a similar job to the one they had at the time (1, very difficult, 4, very easy).

*Perceived career prospects (PCP)*. This one-item measure was specifically developed for the aims of the Professional Paths survey. The respondents were asked to indicate on a four-point scale their agreement with the statement that their overall career prospects were good (1, strongly disagree to 4, strongly agree).

*Background characteristics*. Information on participants’ age, gender (1, male; 2, female), children (1, minor children at home; 2, no minor children at home), and household income at T0 were considered (1, lowest annual income; 8, highest annual income).

### Statistical analyses

The data were analyzed using SPSS and AMOS version 27.0. First, bivariate correlations were calculated to examine the pattern of relationships between the study variables in the promotion and no promotion subsamples. This information was used to evaluate the necessity of including demographic variables as statistical controls in longitudinal analyses as well as to make sure there was no overlap between the investigated outcome variables (i.e., *r* ≥ 0.80). Furthermore, to compare the mean levels of outcome indicators at T0 in the promotion and no promotion subsamples, an independent sample *t*-test analysis was conducted. To inspect the longitudinal dynamics of the outcome variables from T0 to T2, latent growth curve modeling was used.

In the first step, univariate latent growth models were tested to inspect the dynamics of work stress, job satisfaction, perceived career prospects, and perceived employability in each sample. Because our hypotheses focus on the after-effects of a promotion, time was centered at the last timepoint in these analyses. Model fit was assessed using the comparative fit index (CFI) and root mean square error of approximation (RMSEA) on the basis of the recommended cutoff values (CFI > 0.95 indicating excellent fit and CFI > 0.90 indicating acceptable fit, RMSEA < 0.05 indicating excellent fit and RMSEA values between 0.05 and 0.10 indicating acceptable fit) (Hu & Bentler, 1999; MacCallum et al., 1996). A multi-group approach was then used to compare the promotion and no promotion subsamples by comparing a constrained model with a freely estimated model. In the constrained model, the means of the levels and slope factors were constrained to be equal (one at a time) across the two groups, while in the freely estimated model they were allowed to vary. The conclusion about significant differences between the two groups was based on a significant chi-squared difference test.

In the final step, separate latent growth models were tested in subsamples defined by gender and promotion status. Subsample comparisons were conducted using the multi-group approach, as described above.

## Results

On a descriptive level (see Appendix for more details), the following autocorrelations were observed: *r* > 0.60 for general work stress, *r* > 0.28 for job satisfaction, *r* > 0.40 for perceived employability, and *r* > 0.37 for perceived career prospects. The intercorrelations between different outcome variables ranged from negligible (*r* = 0.01) to occasionally strong (*r* = 0.61), but we did not observe a systematic overlap. Furthermore, employees in the promotion sample had significantly lower levels of work stress [*ΔM* = −0.168, *t*(271) = −2.551, *p* = 0.011] and higher levels of perceived career prospects [*ΔM* = 0.317, *t*(271) = 3.390, *p* = 0.001] compared with their counterparts in the non-promotion sample at T0. There were no significant subsample differences regarding the initial levels of job satisfaction [*ΔM* = 0.063, *t*(271) = 0.925, *p* = 0.356] and perceived employability [*ΔM* = 0.176, *t*(271) = 1.850, *p* = 0.065].

Before testing the hypotheses, we also inspected the necessity to include background characteristics as covariates. To this end, all latent growth models were tested with and without the covariates and yielded highly similar results. For the sake of parsimony, we will report only findings from analyses that did not include covariates.

**Figure 1:**
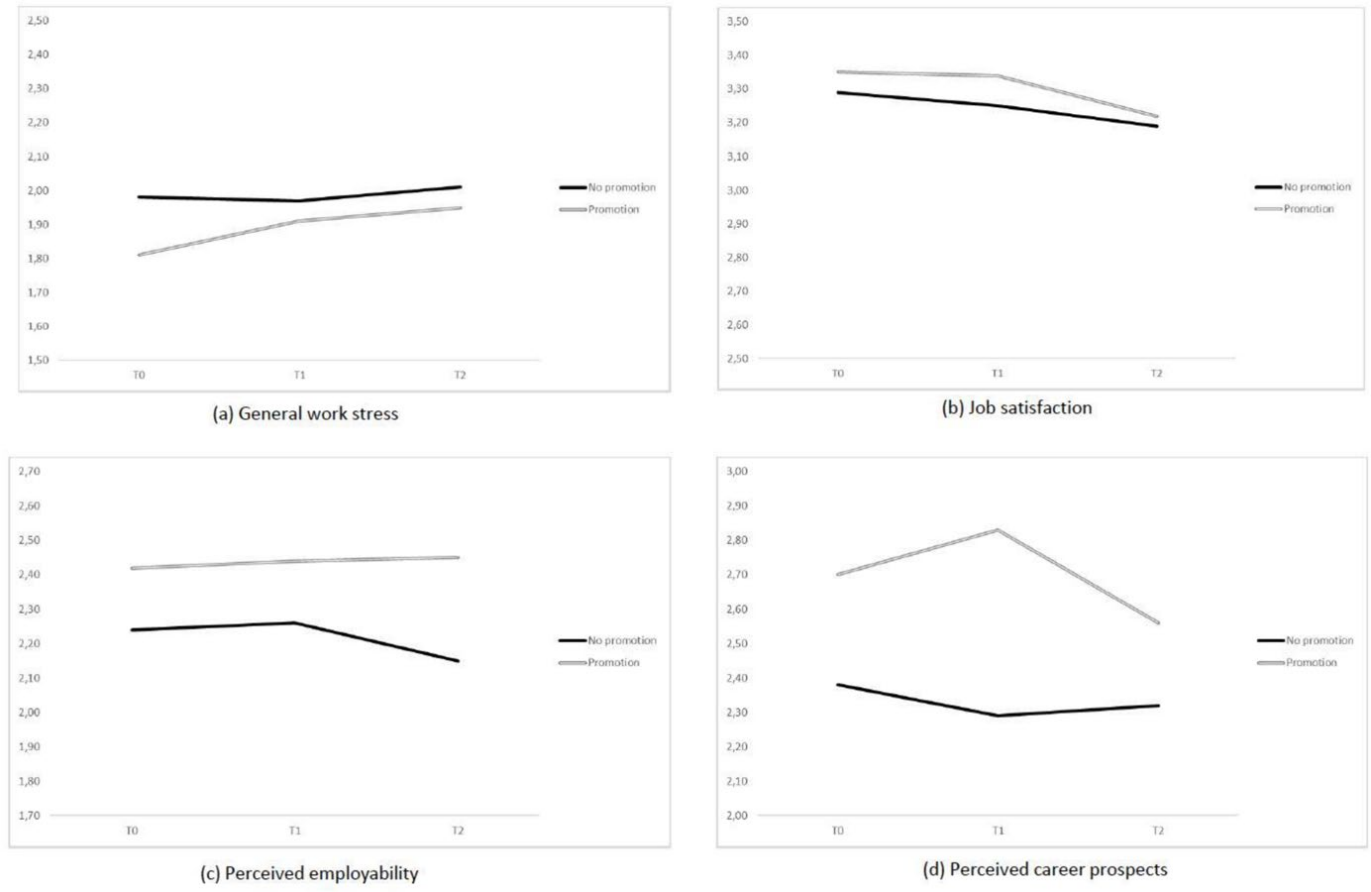
Changes in the outcome variables over time in the promotion and no promotion samples

Table 1 informs about the outcome variables’ growth estimates from T0 to T2 in the promotion versus non-promotion subsamples (see Fig. 1 for a graphical illustration). Model fit was excellent for job satisfaction [*χ*^2^ = 2.525(6), *p* = 0.866, CFI = 1.000, RMSEA < 0.001] and general work stress [*χ*^2^ = 2.716(6), *p* = 0.844, CFI = 1.000, RMSEA < 0.001], and satisfactory for perceived employability [*χ*^2^ = 16.984(6), *p* = 0.009, CFI = 0.960, RMSEA = 0.081] and career prospects [*χ*^2^ = 21.014(6), *p* = 0.002, CFI = 0.932, RMSEA = 0.095]. Formal model comparisons are provided in Table 2. According to the results, the levels and dynamics of job satisfaction were similar in both subsamples, showing a slight but significant decrease over time. The slope of perceived employability was non-significant in both cases. However, it had a positive sign among the promoted employees and a negative sign among those who were not promoted. In other words, perceived employability increased in the promoted subgroup and decreased in the non-promoted subgroup. As a result, in the latter subsample the levels of perceived employability were significantly lower at the end of the study. Work stress showed an increase among the promoted participants only; accordingly, the overall latent growth model (i.e., accounting for the latent intercept and slope) was significantly different between the two subsamples. Regarding perceived career prospects, a decreasing tendency was observed among the promoted employees. However, even though we could not formally test a quadratic (i.e., non-linear) growth model with only three time-points, the growth plot shown in Fig. 1d revealed that the mean levels of this variable increased from T0 to T1 (year of the promotion) and then substantially decreased from T1 to T2 (post-promotion period) in this subsample. By way of contrast, a rather flat pattern was observed in the non-promotion sample.

**Table 1:** Mean intercept and slope factor estimates across different subsamples

Subsamples	By promotion status		By promotion status and gender			
Outcome variables	Promotion (*n* = 128)	No promotion (*n* = 150)	Promotion M (*n* = 63)	Promotion W (*n* = 65)	No promotion M (*n* = 74)	No promotion W (*n* = 76)
Work stress						
Intercept	1.953***	1.998***	1.903***	2.004***	2.043***	1.952***
Linear slope	0.063**	0.011^ns^	0.034^ns^	0.093**	−0.005^ns^	0.024^ns^
Job satisfaction						
Intercept	3.234***	3.189***	3.252***	3.226***	3.165***	3.213***
Linear slope	−0.066*	−0.052*	−0.069^ns^	−0.062^ns^	−0.059^ns^	−0.046^ns^
Employability						
Intercept	2.447***	2.173***	2.367***	2.523***	2.180***	2.167***
Linear slope	0.012^ns^	−0.047^ns^	−0.089^ns^	0.108^ns^	−0.042^ns^	−0.053^ns^
Career prospects						
Intercept	2.625***	2.296***	2.715***	2.537***	2.259***	2.324***
Linear slope	−0.071*	−0.033^ns^	−0.053^ns^	−0.094^ns^	−0.075^ns^	0.002^ns^

For convergence reasons, in conducting promotion status × gender comparisons the slope variance for job satisfaction and career prospects was fixed to a very small value.

*M* men, *W* women, *ns* = non-significant

***p < 0.001, **p < 0.01, *p < 0.05

**Table 2:** Subsample comparison

Compared models	Overall sample	Promotion subsample	No promotion subsample
	Promotion versus no promotion	Men versus women	Men versus women
Work stress			
Intercept	0.347(1)^ns^	0.735(1)^ns^	0.946(1)^ns^
Linear slope	2.820(1)^ns^	1.683(1)^ns^	0.463(1)^ns^
Intercept and slope	6.813(2)*	1.685(2)^ns^	2.752(2)^ns^
Job satisfaction			
Intercept	0.464(1)^ns^	0.022(1)^ns^	0.086(1)^ns^
Linear slope	0.125(1)^ns^	0.009(1)^ns^	0.010(1)^ns^
Intercept and slope	1.500(2)^ns^	0.258(2)^ns^	0.178(2)^ns^
Employability			
Intercept	7.561(1)**	1.047(1)^ns^	0.011(1)^ns^
Linear slope	1.426(1)^ns^	6.096(1)*	0.036(1)^ns^
Intercept and slope	7.834(2)*	6.619(2)*	0.036(2)^ns^
Career prospects			
Intercept	8.083(1)**	1.090(1)^ns^	0.115(1)^ns^
Linear slope	0.161(1)^ns^	0.074(1)^ns^	0.192(1)^ns^
Intercept and slope	23.432(2)***	2.037(2)^ns^	0.193(2)^ns^

The table provides* Δχ*^2^(*df*) estimates after comparing an unconstrained model with a model where the intercept, slope, or all factors are constrained to equality across the compared groups

***p < 0.001, **p < 0.01, *p < 0.05

**Figure 2:**
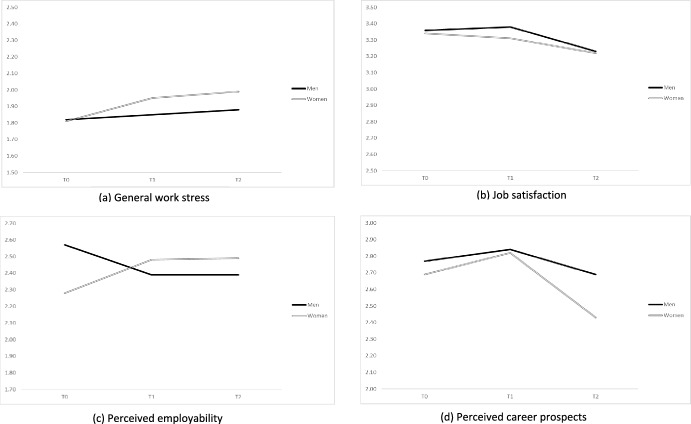
Promotion and changes in the outcome variables over time by gender

Furthermore, results on gender differences are provided in the right section of Table 1 and Table 2 and are graphically illustrated in Fig. 2. As presented in Table 1, none of the outcome variables showed significant dynamics in the male subsample, irrespective of their promotion status. The significant growth patterns of work stress observed in the overall promotion subsample were much more notable among women. The change plot (see Fig. 2d) also revealed a more-pronounced non-linear pattern of career prospects among women. However, formal comparisons (see Table 2) did not support gender differences with regard to these outcomes. The only significant difference between the promoted men and women was observed in the dynamics of perceived employability: a positive (i.e., growing) and a negative (i.e., decreasing) employability slope was found respectively in women and men.

## Discussion

The present study examined the role of promotion in employees’ work-related health and happiness in terms of work stress and job satisfaction, and their career-related performance in terms of perceived employability and career prospects using a three-wave design. Specifically, we investigated the changes in promoted employees’ sustainable career development outcomes and compared them with a sample of employees who did not get a promotion during the entire time of the study. We then tested these relations among men and women separately. By doing so, our main aim was to expand our understanding of the consequences of a career event on employees’ health, happiness, and productivity, the three core indicators of career sustainability. Our findings shed light on both positive and negative effects of the promotion on the investigated career sustainability indicators over time. All the indicators were considered together and by taking the time and gender dimensions into account, results showed that promotion does not necessarily foster career sustainability, neither for men nor for women.

### After a promotion: lower level of job satisfaction and higher level of work stress

We expected that the promotion sample would report a higher levels of job satisfaction without a persistent linear increase in it, while in the non-promoted sample, job satisfaction would remain stable through the three waves. Surprisingly, this hypothesis (*H1a*) was not supported. Both samples showed quite similar patterns of results from the pre-promotion to the post-promotion periods. Indeed, job satisfaction slightly decreased over time for all study participants. Such results may be explained by the prevailing trends in the current labor market, which is more and more precarious, making people less satisfied with their working conditions (Lopes et al., 2014). Moreover, one study showed recently that working for the same company for many years makes people less satisfied with their job regardless of their age (Dobrow et al., 2018). In our study, we observed a very low rate of job changes. This means that individuals were in general working for the same company for many years, which may explain also why job satisfaction was decreasing over time. However, we could still expect that getting a promotion could at least temporarily prevent the decrease in job satisfaction as postulated in the honeymoon-hangover effect (Boswell et al., 2005). One possible explanation behind the non-significant effect of promotion on job satisfaction could be due to statistical power issues, as the investigated sample was relatively small. It is also possible that a 1-year time lag between the measurements was not ideal for capturing the hypothesized effects (some participants could have been promoted a few days before the study, whereas others could have experienced a promotion almost a year before). Therefore, the immediate consequences of a promotion on job satisfaction could have been not well captured with this method. Finally, yet another explanation could be related to the Swiss labor market context that offers quite stable and secure employments where individuals feel quite satisfied with their working conditions according to the Organization for Economic Cooperation and Development (OECD, 2020). This could explain why getting promoted may not have such a powerful impact as it would have in less privileged contexts.

Concerning work stress, we expected the promoted individuals to report higher and more persistent increase in work stress from the pre-promotion to post-promotion period compared with the non-promoted ones. This hypothesis (*H1b*) was confirmed. Indeed, our results align to the findings previously reported in the literature (Boyce & Oswald, 2012; Johnston & Lee, 2013; Nyberg et al., 2017). This suggests that a normatively considered positive event does not necessarily have only positive impact on one’s life. Promotion could also create more stress at work, especially among women according to our results. Multiple reasons have been already evoked in the literature to explain the negative impact of career progression among women (Coronel et al., 2010; Nyberg et al., 2015, 2017). For example, we can point out the difficulties to manage work and family, especially in Switzerland where it is expected that women take care of children and household responsibilities, often leading them to accept a part-time job (Federal Statistical Office, 2019; Levy et al., 2006). They could feel more stressed at work because they have higher responsibilities and more demands that are more difficult to fulfill when additional non-work responsibilities must be taken in parallel (Nyberg et al., 2015). In relation to this, the organizational work culture could act as a barrier and contribute to the stress felt at the workplace (Coronel et al., 2010). Finally, in light of gender inequalities at work (e.g., in terms of salary, managerial positions), women may experience heightened pressure after getting promoted, as they strive to demonstrate that they can take responsibilities and perform their duties as well as men.

Finally, in our study, the promoted sample had a very low stress level at T0 compared with the non-promoted sample, and 1 year after the promotion both samples showed the same level of stress. It is possible that promoted employees, while initially exhibiting lower levels of stress, may not have possessed the optimal managerial skills required for higher-level positions. Hence, our study could be another evidence for the Peter Principle (Benson et al., 2019).

### Perceived employability after a promotion: not the same for men and women

We expected perceived employability to increase from the pre-promotion to post-promotion period among the promoted employees, while remaining stable among the non-promoted ones (*H2*). This hypothesis was not supported. We found no evidence for an effect of promotion on the growth of perceived employability over time. However, the initial level of employability was different across the compared samples. Indeed, promoted employees showed higher initial levels of perceived employability than the non-promoted ones. Employability may serve as a resource that facilitates promotion and acts as an antecedent, as pointed out in the literature (e.g., Andresen et al., 2022).

Although no differences were observed between the promoted and non-promoted samples overall, we found some gender differences in this outcome. Indeed, a noticeable increase in perceived employability among women following a promotion and a decline in perceived employability among men following a promotion was observed. These findings suggest that promotion may act as a career booster for women. Such findings are rather surprising, but they may be due to different patterns of advancement men and women were typically subjected to. Although we did not directly measure this factor in our study, it is plausible that men were more likely to be promoted to executive positions, which inherently limits the number of comparable positions in the labor market (hence, leading to lower perceived employability). Notably, this would not hold true for lower-level promotions, which can potentially open up good possibilities for further advancement.

### After a promotion: higher level of career prospects, but only temporarily

We expected perceived career prospects to increase from the pre-promotion to post-promotion period among the promoted employees, while remaining stable among the non-promoted ones (*H3*). This hypothesis was not fully supported. Indeed, on the basis of our graphical findings, there seemed to be a curvilinear effect among the promoted participants (although we could not formally test it), but no such dynamics were observed in the non-promoted sample. Hence, promotion seems to have a short-term positive impact on perceived career prospect among the promoted ones and this effect significantly declined 1 year after, resulting in the overall negative dynamics. Moreover, the promoted employees not only showed higher levels of perceived career prospects at T2, but their initial levels (i.e., before getting a promotion) were also higher compared with the non-promoted employees. One possible explanation for this difference could be that their promotion was anticipated or foreseen, thus individuals perceived favorable opportunities for advancement prior to their actual promotion. This could also explain why 1 year later the levels of career prospects dropped among the promoted ones, reaching their scented maximum level, and therefore no longer perceiving any opportunities for promotion or advancement.

As seen in the plotted output, the curvilinear effect appeared to be somewhat more pronounced for women compared with men, despite the formal analysis revealing no significant differences (which may be due to a relatively small sample, and thus lower power to detect subtle differences). Getting promoted for women is a work event that could give more confidence in one’s ability to perform in a given task and help to see in a positive way at least temporarily their career future knowing that they have more difficulties than men in reaching higher positions (International Labour Office, 2020).

### Implication for career sustainability development

One of the aims of this article was to clarify the role of work-related events in sustainable career development, especially the role of positive work events that were shown to foster career sustainability in previous research (Udayar et al., 2021). In this study, by focusing on one specific positive event, namely promotion, and by taking into account the time and gender dimensions, we found no clear evidence that promotion would foster career sustainability, although it seems to foster career-related performance indicators for women. At the same time, we observed a hindering effect of promotion on perceived stress at work (considered a health indicator of sustainability in the current study) and we also found that job satisfaction (a happiness indicator) continued to decrease despite the promotion. Through the comprehensive examination of multiple indicators of career sustainability and the adoption of a broad integrative perspective, our findings lead us to the conclusion that a positive career event, such as promotion, does not necessarily contribute to a sustainable career irrespective of the participant’s gender.

Moreover, this study showed the importance of considering the perspective of different demographic groups (i.e., gender) when studying career sustainability and especially when exploring the impact of various career events or changes on perceived health, happiness, and career-related performance. While previous meta-analyses have indicated a non-significant effect of gender on health, happiness, and performance (e.g., Batz-Barbarich et al., 2018; Harari et al., 2021; Purvanova & Muros, 2010), our study showed that in some cases women and men could react differently to important work events, and this could result in different dynamics in their career development. The career sustainability framework should emphasize more the moderating role of sociodemographic characteristics when analyzing the interaction between the person and context and their impact of career sustainability development indicators. Currently, the framework puts in the center the person with their capacity to adapt, be proactive, and create meaning (De Vos et al., 2020), but it should consider the person as a whole and take into account a person’s background characteristics, which could determine the way they adapt to a situation and make meaning of it.

Finally, this study highlights the importance of understanding the temporal dynamics of the consequences of a work event on health, happiness, and performance, as well as using different time lags within the same study. Indeed, employing a pre-, during, and post-promotion design and differentiating between the year of the promotion and 1 year after helped to better understand the role of time dimension in moderating the impact of promotions on the three indicators of career sustainability.

### Limitations and suggestions for future research

The present study has several limitations that have to be taken into account when interpreting the findings. First, while we managed to spot some after-effects of the promotion, our study was based on three measurement points only. This may be not enough for detecting longer-term consequences of a career advancement and especially the changes in the valence of the promotion per se as compared with its outcomes. Therefore, we would recommend that future longitudinal studies focus on longer time lags, also potentially investigating a wider range of employee well-being outcomes. It would be particularly useful to study the different aspects of outcomes more in detail, for instance, separating between narrow (such as work engagement, role clarity, etc.) versus global (such as overall job satisfaction) indicators of well-being, as well as identifying their dynamics over time. Second, although the design of our study was specifically aimed at testing pre-, during, and post-promotion experiences, it is possible that we did not spot the actual moment of the promotion. The participants of our study were measured on a yearly basis and the actual event of promotion may have taken place any time during the preceding year. For this reason, the observed effects might be weaker than they really are and future studies that are interested in the dynamics of the career advancement experience may adopt a more dynamic approach (e.g., by using a diary design), which should be combined with a panel study approach. In addition, the 3-year period analyzed here does not start in the same year for all participants. The general context of the years taken into consideration could therefore vary from one participant to another.

Another factor that should be considered in future studies on promotions is the distinction between expected, planned, and wanted/desired promotions versus unexpected, unplanned, and unwanted ones, which could act as career shocks and have a stronger effect on career sustainability.

Another interesting entry point for future study on promotion is to compare people who are at the beginning of their career with those who are in the middle or approaching the end of their careers, which was not possible to do in our study due to the small sample size. A promotion could be experienced differently depending on the career stage one is at.

Finally, our findings provide only a brief insight into the gender differences regarding the subjective experience of promotion. The compared groups were rather modest in size, which somewhat limits the power of the comparisons and encumbers the interpretation of results. We therefore suggest that future comparative studies employ larger sample sizes. Additionally, other potential boundary conditions that may determine the outcomes of the promotion should be investigated as they are largely under-explored in this area of research.

## Conclusions

The present study examined the role of promotion in employees’ career sustainability using a three-wave design. The results pointed out both positive and negative effects of promotion on employees health (i.e., work stress), happiness (i.e., job satisfaction), and career-related performance (i.e., perceived employability and career prospects). These findings call into question the shared view that promotion is unequivocally beneficial for the workers, and ultimately highlights further perspectives for investigating the role of career events in sustainable career development. Moreover, the present study adds to the literature by discussing the role of gender in moderating the effect of a promotion on the three indicators of a sustainable career.

In terms of practical implications, the findings suggest that organizations should be aware that career mobility through a job promotion may have detrimental consequences on workers’ health, happiness, and productivity, preventing them from developing a sustainable career. To promote career sustainability for all, they should give support and training on how to manage such transitions. Moreover, organizations should implement gender inequality awareness measure and training for all workers. Understanding gender issues, as well as raising awareness of gender disparity, is crucial for starting a structural reform process that will improve gender equality inside an organization and promote a sustainable career development for all.

In parallel, at the individual level, educational and vocational guidance should also address those issues. Career counselors should be first aware that some social groups may be more at risk of developing a sustainable career, such as women, especially in Switzerland. They may then help those women in an informed way and use critical consciousness (Cadenas & McWhirter, 2022) to make them realize barriers to career sustainability and engage in action to overcome those barriers.

## Appendix: Variable means, standard deviations, and correlations in the promotion and no promotion samples

**Table:**

Variables	Mean (*SD*)		1	2	3	4	5	6	7	8
	Promotion sample	No promotion sample								
1. Age	41.02 (8.70)	41.76 (7.88)	–	0.19*	0.01	−0.01	−0.09	0.04	−0.20*	−0.07
2. Gender	1.51 (0.50)	1.51 (0.50)	0.20*	–	0.06	−0.05	−0.10	0.03	−0.01	−0.07
3. Children	1.31 (0.47)	1.28 (0.45)	0.20	0.10	–	−0.21*	0.04	−0.15	−0.02	−0.09
4. Income	5.35 (2.04)	4.89 (1.93)	0.01	−0.13	−0.16	–	−0.03	0.05	0.10	0.09
5. GWS (T0)	1.81 (0.50)	1.98 (0.58)	0.11	−0.03	0.13	0.07	–	−0.46***	−0.16	−0.25**
6. JS (T0)	3.35 (0.53)	3.29 (0.60)	−0.03	−0.02	−0.04	−0.01	−0.25**	–	0.07	0.38***
7. PE (T0)	2.42 (079)	2.24 (0.77)	−0.21*	−0.19*	−0.14	0.02	−0.03	0.11	–	0.15
8. PCP (T0)	2.70 (0.76)	2.38 (0.78)	−0.08	−0.09	−0.13	0.01	−0.15	0.23*	0.05	–
9. GWS (T1)	1.91 (0.58)	1.97 (0.56)	0.06	0.08	0.15	0.01	0.69***	−0.31**	−0.05	−0.15
10. JS (T1)	3.34 (0.57)	3.25 (0.59)	0.07	−0.07	−0.11	−0.07	−0.29**	0.35***	0.16	0.15
11. PE (T1)	2.44 (0.79)	2.26 (0.78)	−0.32***	0.05	−0.24*	−0.04	0.08	< 0.01	0.47***	0.09
12. PCP (T1)	2.83 (0.67)	2.29 (0.77)	−0.13	−0.01	−0.18	−0.18*	−0.18*	0.08	−0.05	0.37***
13. GWS (T2)	1.95 (0.67)	2.01 (0.59)	0.01	0.06	0.25*	−0.05	0.65***	−0.14	−0.03	−0.11
14. JS (T2)	3.22 (0.61)	3.19 (0.62)	0.11	−0.01	−0.13	−0.04	−0.28**	0.28**	0.09	0.13
15. PE (T2)	2.45 (0.85)	2.15 (0.77)	−0.21*	0.06	−0.11	< 0.01	−0.03	0.04	0.40***	0.13
16. PCP (T2)	2.56 (0.73)	2.32 (0.72)	−0.12	−0.16	−0.12	−0.05	−0.26**	0.21*	0.21*	0.44***

Variables	Mean (*SD*)		9	10	11	12	13	14	15	16
	Promotion sample	No promotion sample								
1. Age	41.02 (8.70)	41.76 (7.88)	−0.05	0.13	−0.38***	−0.03	0.01	0.10	−0.31***	0.02
2. Gender	1.51 (0.50)	1.51 (0.50)	−0.17*	0.03	0.04	0.01	−0.04	0.04	−0.03	0.02
3. Children	1.31 (0.47)	1.28 (0.45)	0.05	−0.05	0.01	−0.07	0.12	−0.06	−0.12	−0.07
4. Income	5.35 (2.04)	4.89 (1.93)	−0.08	0.06	−0.02	0.03	0.01	−0.06	−0.07	−0.06
5. GWS (T0)	1.81 (0.50)	1.98 (0.58)	0.62***	−0.23**	−0.06	−0.15	0.61***	−0.16*	−0.17*	−0.21*
6. JS (T0)	3.35 (0.53)	3.29 (0.60)	−0.38***	0.53***	0.07	0.34***	−0.44***	0.51***	0.13	0.43***
7. PE (T0)	2.42 (079)	2.24 (0.77)	−0.12	0.11	0.56***	0.01	−0.15	−0.01	0.57***	0.09
8. PCP (T0)	2.70 (0.76)	2.38 (0.78)	−0.15	0.29***	0.20*	0.56***	−0.07	0.31***	0.23**	0.68***
9. GWS (T1)	1.91 (0.58)	1.97 (0.56)	–	−0.39***	−0.10	−0.15	0.67***	−0.23**	−0.14	−0.15
10. JS (T1)	3.34 (0.57)	3.25 (0.59)	−0.49***	–	0.14	0.30***	−0.31***	0.56***	0.14	0.29***
11. PE (T1)	2.44 (0.79)	2.26 (0.78)	−0.02	0.01	–	0.24**	−0.09	−0.02	0.70***	0.14
12. PCP (T1)	2.83 (0.67)	2.29 (0.77)	−0.24**	0.34***	0.14	–	−0.08	0.31***	0.12	0.61***
13. GWS (T2)	1.95 (0.67)	2.01 (0.59)	0.80***	−0.33***	−0.05	−0.12	–	−0.37***	−0.14	−0.24**
14. JS (T2)	3.22 (0.61)	3.19 (0.62)	−0.42***	0.48***	0.03	0.25**	0.49***	–	−0.02	0.46***
15. PE (T2)	2.45 (0.85)	2.15 (0.77)	−0.13	0.01	0.70***	0.12	−0.12	0.02	–	0.17*
16. PCP (T2)	2.56 (0.73)	2.32 (0.72)	−0.35***	0.24**	0.21*	0.51***	−0.28**	0.25**	0.27**	–

Correlations in the promotion sample (*n* = 128) are presented below the diagonal. Correlations in the no promotion sample (*n* = 150) are presented above the diagonal.

*GWS* general work stress, *JS* job satisfaction, *PE* perceived employability, *PCP* perceived career prospects

****p* < 0.001, ***p* < 0.01, **p* < 0.05
